# Concurrent Validity of Digital Vascular Auscultation for the Assessment of Blood Flow Obliteration on the Radial Artery in Healthy Subjects

**DOI:** 10.3390/diagnostics10070494

**Published:** 2020-07-18

**Authors:** María-Dolores Cortés-Vega, María Jesús Casuso-Holgado, Ángel Oliva-Pascual-Vaca, María-Isabel García-Bernal, Paula González-García, Cleofás Rodríguez-Blanco

**Affiliations:** Department of Physiotherapy, Faculty of Nursing, Physiotherapy and Podiatry, University of Seville, C/Avicena s/n, 41009 Seville, Spain; mdcortes@us.es (M.-D.C.-V.); angeloliva@us.es (Á.O.-P.-V.); ibernal@us.es (M.-I.G.-B.); pgonzalez@us.es (P.G.-G.); cleofas@us.es (C.R.-B.)

**Keywords:** stethoscope, Doppler, auscultation, physical therapy, thoracic outlet syndrome

## Abstract

This study aimed to determine the validity of digital vascular auscultation for the assessment of changes in the radial pulse in healthy subjects, using Doppler sonography as a validated test referent. Sixty-one non-symptomatic subjects (mean age of 52.5 ± 16.1 years) were assigned and evaluated under one of the following conditions: In condition 1, blood flow of the radial artery was not modified; for condition 2, blood flow of the radial artery was modified using a pressure sleeve around the humerus. The radial pulse was then measured three times with each diagnostic tool by three different blinded evaluators. Both instruments demonstrated a high association between the identification of blood flow modifications or not and the assigned condition (*p* < 0.001). A strong concordance between the two devices when detecting the “changes” or “no changes” in blood flow was demonstrated (*k* = 0.936, *p* < 0.001). Stethoscope sensitivity was 95%, and specificity was 99%. In conclusion, digital vascular auscultation seems to be a valid technique to examine blood flow changes of the radial artery in non-symptomatic subjects, and it could be useful for physical therapists when combined with provocative tests for the screening of possible thoracic outlet syndrome in patients.

## 1. Introduction

Thoracic outlet syndrome (TOS) constitutes a group of diverse disorders, with different aetiologies, that trigger the compression of one or more neurovascular elements as they traverse the thoracic outlet [1]. Moreover, neurovascular compression can potentially occur at three different anatomic levels: the interscalene triangle, the costoclavicular space, or the pectoralis minor space. Thus, TOS can be defined as a neurovascular syndrome associated with compression of the brachial bundle (brachial plexus and/or subclavian vessels) [2].

Non-specific TOS, also named disputed or symptomatic TOS, is the most prevalent subtype of this syndrome; although in most cases the literature refers to this type as neurogenic TOS [3]. The most frequently reported symptoms are pain, paresthesia, and loss of muscle strength associated with a muscular dysfunction [1]. In this subtype of TOS, physical therapy interventions are highly recommended [4].

The diagnosis of TOS is usually accomplished by careful examination of medical history, physical examination (irritant examination), radiological examination, and electrodiagnostic examination [5]. However, because of the complex structure of the thoracic outlet, diagnosis of TOS is still a challenge.

The performance of provocative manoeuvres is indicated for the clinical diagnosis of neurogenic TOS [6,7]. Adson’s test, Eden’s test, Wright’s test, and the Roos or Elevated Arm Stress Test (EAST) are commonly used for this purpose. They are performed during palpation of the radial pulse, and they are positive when neurological peripheral symptoms are reproduced and total or partial obliteration of the radial pulse occurs [8].

Nevertheless, the diagnostic accuracy of these clinical tests is controversial [9]. A recent systematic review observed poor evidence in support of the validity of clinical tests for the diagnosis of TOS, and it is not clear which of the tests has the highest diagnostic accuracy [10]. However, studies have indicated that reliance on multiple tests in conjunction may increase diagnostic accuracy [11]. Guillard et al. [12] demonstrated that all pairs of tests, including Adson’s test, were significantly correlated with the final diagnosis of TOS. Furthermore, when five tests were positive, sensitivity and specificity were each 84%.

Physical therapists have an important role in the screening of TOS patients. In a retrospective cohort study of 621 patients referred to a medical institution for further imaging diagnosis, it was observed that 91% of patients referred by a physical therapist were finally diagnosed with TOS [13]. Therefore, it is important for physical therapists to have accurate screening tools.

Although there is currently no true gold standard for the diagnosis of TOS, in combination with a clinical assessment, the use of Doppler sonography imaging in a provocative position can be considered a reference standard [10,14]. Changes in blood flow for the radial artery or compression of the subclavian vessels during arm positioning similar to clinical tests can be examined [10,15].

It is known that Doppler sonography is a valid and reliable clinical tool for peripheral artery evaluation [16,17,18]; however, it is costly and unavailable for most physical therapists. Therefore, it is important to validate more inexpensive tools for this purpose. In this sense, the stethoscope could be an alternative evaluation tool for the assessment of peripheral blood flow changes related to TOS. It is affordable, easy to use, and has been widely recognised as a valid diagnostic method for cardiac auscultation [19,20] and peripheral arterial disease [21,22,23]. Furthermore, it has demonstrated a higher accuracy for radial artery pulse assessment than the palpatory method [24].

This paper evaluates a method for detecting occluded blood flow in the upper extremity of subjects without neurovascular symptoms using a digital stethoscope. The aim of this research was to investigate the validity of digital vascular auscultation for the detection of partial blood flow obliteration of the radial artery in asymptomatic TOS subjects, using Doppler sonography as a validated test reference. This procedure could be considered a previous step for the use of this method in the detection of blood flow changes related to TOS.

## 2. Materials and Methods

### 2.1. Subjects

A cross-sectional validation study was conducted with a repeated clinical measurements design. A sample of 71 subjects was recruited at the University of Sevilla (Spain). Inclusion criteria were the following: adults of both genders from 20–70 years of age without the presence of neurovascular symptoms in the upper limbs. Exclusion criteria were the following: surgery on the costoclavicular joint, shoulder girdle, elbow, or wrist; muscle pain in the region of the neck, shoulder, and arm; presence of any acute disease; and the existence of psychiatric disorders.

This study was approved by the Experimental Research Ethics Committee of the University of Sevilla (20 March 2011 approved). Written informed consent was obtained from each participant before inclusion. In addition, all procedures were performed in accordance with the Declaration of Helsinki. This research was performed following the recommendations of the Quality Assessment of Diagnostic Accuracy Studies tool (QUADAS-2) [25] and the Consensus-based Standards for the Selection of Health Measurement Instruments (COSMIN) [26].

### 2.2. Instruments

The equipment used consisted of an MX3 Plus sphygmomanometer (Omron, HEM-742-E, Japan), MDR-XB 500 headphones (Sony, MDR-XB 500, Tokio, Japan), a 3M Littmann model 3200 digital stethoscope (Littmann 3200, London, ON, Canada), and an Elcat model Handydop^®^-Pro Doppler (Elcat, Handydop Pro, Wolfratshausen, Germany) connected to a laptop via Handydop Pro Vasoview software. Participants reported to an experimental laboratory for approximately 1 h.

### 2.3. Procedure

First, for each participant, the main researcher evaluated the anthropometric measurements of weight, height, and blood pressure, and filled out a form related to cardiovascular risk factors of the subject (hypertension, diabetes, and tobacco consumption). Subjects were then randomly allocated into two assessment conditions using a random number generator tool. Condition 1 consisted of no modification of the blood of the upper limbs. In contrast, condition 2 consisted of modification of blood flow pressure by a sphygmomanometer placed on the right brachial artery at the level of the humerus without interposition of clothing. The pressure increased by up to 20 mmHg above the previously measured systolic pressure [27]. The main researcher was the only person who knew the group allocation.

**Figure 1:**
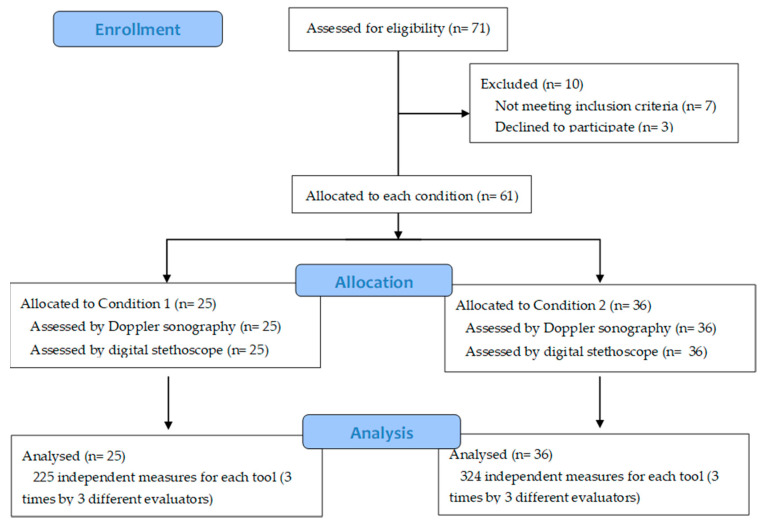
Study flow chart.

Three independent examiners carried out both procedures (digital stethoscope and Doppler sonography) on each subject. Prior to the study, all examiners were trained at using the two diagnostic devices. This way, the radial pulse of the 61 subjects was measured 3 times by 3 different evaluators, with a total of 549 measures for each diagnostic tool (Figure 1). In order to blind the evaluators, a cabin isolated from the exterior by a heavy curtain was designed. Use of the curtain allowed us to divide the participant’s body into two working areas—the upper part, which was inside the cabin where the main investigator was modifying the brachial artery pressure (in the case of condition 2) and registering the vascular blood flow changes reported by the examiner, and the lower part, with the examiner standing by the subject’s feet (outside the cabin). In addition to being visually concealed from the subject, the examiners were also acoustically concealed with the use of headphones connected to either the Doppler device or to the digital stethoscope, according to the case. Thus, the examiners were completely blinded to the modification (or not) of the pressure applied to the artery by the main researcher.

#### Assessment

Participants were placed in supine decubitus and relaxed with their right arm resting along the side of their body and their forearm and wrist in a neutral position, to allow full exposure of the artery for palpation. In all cases, evaluation of the radial pulse was performed on the right arm [27].

Once the subject was ready, the first examiner proceeded to the room and used a dermographic marker to mark the reference point over the radial artery at which the evaluation was to be carried out, to ensure that it was the same in each subsequent measurement (approximately 2 centimetres above the radial styloid on the palmar side of the forearm). Digital stethoscope measurements were done first. Once the examiner was ready and heard the pulse through the stethoscope headphones, the main researcher indicated the beginning of the measurement by a visual signal over the heavy curtain. Changes in the radial pulse were then observed for a 10 s time interval. If any change in blood flow was detected, the examiner immediately reported it to the researcher, who registered the event as a “change”. Each measurement was made three times by the same examiner, leaving one minute for a washout of the explorations of the radial pulse to avoid disturbances by mechanical stress at the vascular level [28]. After the third repeated measurement, the examiner left the room and the second examiner repeated the same procedure with the same subject. Therefore, no two examiners were present at the same time in the intervention room. Once the three examiners finished their measurements, the same procedure used previously was repeated, but with the use of Doppler sonography, and the examiners did not know if they were with the previous subject or with a new subject. The procedures were performed under similar environmental conditions for all subjects.

### 2.4. Statistical Analysis

First, data was grouped into patient categories and screened for any errors, anomalies, or duplications within the set. Baseline sample characteristics (conditions 1 and 2) were compared using the chi-square test. The degree of association between changes in blood flow and assessment condition was determined using an exact Fisher Test. The agreement between the different categories was measured by Fleiss’ kappa index. The validity of the two instruments was verified using contingency tables and by calculating the sensitivity and specificity.

A statistical significance level of 95% (*p* < 0.05) was adopted. All calculations were conducted using the statistical software package SPSS version 18.0 (SPSS Science, Chicago, IL, USA).

## 3. Results

A total of 71 subjects were assessed for eligibility; seven did not meet inclusion criteria and three refused to participate. Finally, a total of 61 participants (18 men and 43 women) were recruited and completed the different evaluations. The average age of the total sample was 52 years (mean age of 52.5 ± 16.1 years). Of the subjects, 41% were randomly assigned to condition 1 and 59% were assigned to condition 2. No differences between groups were observed at baseline. A more detailed description of the sample characteristics is reported in Table 1.

Each instrument demonstrated a high association between the identification of blood flow modifications and the assessment condition. As can be observed in Table 2, changes in blood flow were correctly classified by both devices (*p* < 0.001). A strong concordance (*k* = 0.966, *p* < 0.001) between the Doppler device and digital stethoscope when detecting the “changes” or “no changes” in blood flow was also observed (Table 3).

The probability that cases of “change” in blood flow were correctly classified by the stethoscope in the first 10 s of evaluation was 95% (Se = 0.95; Table 4). This means that 95% of the cases recognised as positive with the Doppler device were also picked up using the stethoscope. Similarly, the probability of correctly classifying cases of “no change” during the first 10 s of evaluation was 99% (Sp = 0.99). The positive predictive value observed (PPV = 0.99) means that the probability that a positive stethoscope result was a case of “change” was 99%. Similarly, the negative predictive value observed (NPV = 0.93) means that the likelihood that a negative stethoscope result was really a case of “no change” was 93%.

With respect to the observed value of the positive likelihood ratio (LR+ = 95), this means that a positive result (registering a “change”) with the stethoscope is 95 times more likely in cases in which there is a 20 mmHg blockage of blood flow at the level of the radial artery than in other cases in which there is no such obstruction. The negative likelihood ratio (LR− = 0.05) means that a negative result with the stethoscope (“no change”) is 0.05 times as likely in cases in which there is actually a 20 mmHg obstruction of blood flow than in others in which there is no such obstruction (Table 4).

## 4. Discussion

This study aimed to examine the validity of using a digital stethoscope for the identification of radial artery blood flow alterations in asymptomatic TOS subjects when compared to Doppler sonography. Our results suggest that digital vascular auscultation can be considered a valid tool for radial artery haemodynamic exploration. We observed that for a pressure of 20 mmHg over the radial artery, there was a high concordance between the two evaluation methods in the first 10 s of exploration (*k* = 0.936).

Our results agree with previous research. Chesbro et al. [29] analysed the correlation between Doppler sonography and use of a stethoscope for the assessment of the ankle-brachial index (ABI) in healthy subjects. The authors observed moderate to very strong agreement between a dual earpiece standard stethoscope and Doppler device when assessing systolic blood pressure in the arm. Conversely, this agreement was moderate to weak for the lower limbs, and for this reason a Doppler ultrasound was recommended over a stethoscope. Similarly, Carmo et al. [22] reported good correlation between use of a stethoscope and Doppler device for ABI measures, with a diagnostic accuracy of the stethoscope of 87.7%.

Conversely, our results are in disagreement with Kaudmann et al. [30], who observed that the coefficients of concordance between the stethoscope and Doppler device for femoropopliteal stenosis identification were low (*k* < 0.3). Similarly, Takahashi et al. [21] concluded that auscultation was not a valid method for peripheral artery disease identification based on ABI measurements, but it could be a clinically useful tool for excluding this disease.

On the other hand, we know that when Doppler ultrasonography is added to provocative clinical tests for TOS diagnosis, a significant gain in specificity is obtained (two provocative tests, Sp = 6%; two provocative tests plus Doppler sonography, Sp = 89%) [12]. Taking into account our results, an estimation of the validity of digital vascular auscultation for TOS identification could be suggested. However, further research in symptomatic patients is needed.

This study has some limitations. First, testing of the reliability of the stethoscope was not performed in our study. However, the interrater reliability of a radial pulse evaluation with a stethoscope has been previously demonstrated to be high [24,29]; Second, the number of measures in each study condition (blood flow modification or not) was not equal. However, this was due to the number of subjects randomly assigned to each condition; Third, we studied the validity of using a digital stethoscope in subjects without the presence of neurovascular symptoms in the upper limbs. Nevertheless, we believe that this procedure could be considered a previous step for the use of this method in the detection of blood flow changes related to TOS.

## 5. Conclusions

Our results show that digital vascular auscultation is a valid technique to examine blood flow interruption of the radial artery in non-symptomatic TOS subjects. It is also an affordable tool that could be useful for physical therapists when combined with provocative tests for the screening of possible TOS patients.

## Figures and Tables

**Table 1:** Characteristics of the study groups and between-group comparisons.

Sample Characteristics		Study Group		Total	Chi-Squared	*p*-Value
		Condition 1	Condition 2			
Gender	Male	8	10	18	0.126	0.722
	Female	17	26	43		
Aged	20–29	3	7	10	2.622	0.758
	30–39	1	2	3		
	40–49	4	5	9		
	50–59	2	6	8		
	60–69	13	15	28		
	70–79	2	1	3		
Handedness	Right	23	34	57	0.114	0.704
	Left	2	2	4		
Tobacco consumption	Smoker	3	3	6	1.740	0.419
	Former smoker	4	11	15		
	Non-smoker	18	22	40		
Treatment for Hypertension	Yes	13	17	30	0.135	0.714
	No	12	19	31		
Diabetes	Yes	4	7	11	0.118	0.731
	No	21	29	50		
Hypertension non-diabetic(BP of [140/90] mmHg)	Yes	13	16	29	0.338	0.561
	No	12	20	32		
Hypertension diabetic(BP of [130/80] mmHg)	Yes	4	6	10	0.005	0.945
	No	21	30	51		

BP: blood pressure.

**Table 2:** Association between changes in blood flow in the first 10 s and assessment condition for Doppler instrument and vascular auscultation.

Tool		Study Group		*p*-Value
		Condition 1	Condition 2	
Doppler	No change	220	1	<0.0001
	Change	5	323	
Stethoscope	No change	224	12	<0.0001
	Change	1	312	

**Table 3:** Levels of concordance between the “changes” and the “no changes” detected by the Doppler instrument and by vascular auscultation in the first 10 s.

Tool		Doppler		Total	Kappa	ASE	*p*-Value
		No Change	Change				
Stethoscope	No change	220 (40.1%)	16 (2.9%)	236	0.936	0.015	<0.0001
	Change	1 (2%)	312 (56.8%)	313			
Total		221 (40.3%)	328 (59.7%)	549			

ASE: Asymptotic standard error.

**Table 4:** Relationship between the detection (or not) of changes in blood flow with the Doppler instrument and with the stethoscope in the first 10 s of examination.

Tool			Doppler		
			Yes	No	Total
Stethoscope		Yes	312	1	313
		No	16	220	236
		Total	328	221	549
Se 0.95 (95%)	Sp 0.99 (99%)	PPV 0.99 (99%)	NPV 0.93 (93%)	LR+ 95	LR− 0.05

Se: sensitivity; Sp: specificity; PPV: positive predictive value; NPV: negative predictive value; LR+: positive likelihood ratio; LR−: negative likelihood ratio.

## References

[1] Laulan J., Fouquet B., Rodaix C., Jauffret P., Roquelaure Y., Descatha A. (2011). Thoracic outlet syndrome: Definition, aetiological factors, diagnosis, management and occupational impact. J. Occup. Rehabil..

[2] Illig K.A., Donahue D., Duncan A., Freischlag J., Gelabert H., Johansen K., Jordan S., Sanders R., Thompson R. (2016). Reporting standards of the Society for Vascular Surgery for thoracic outlet syndrome. J. Vasc. Surg..

[3] Ferrante M.A., Ferrante N.D. (2017). The thoracic outlet syndromes: Part 1. Overview of the thoracic outlet syndromes and review of true neurogenic thoracic outlet syndrome. Muscle Nerve.

[4] Ferrante M.A., Ferrante N.D. (2017). The thoracic outlet syndromes: Part 2. The Arterial, Venous, Neurovascular and Disputed Thoracic Outlet Syndrome. Muscle Nerve.

[5] Zhang T., Xu Z., Chen J., Liu Z., Wang T., Hu Y., Shen L., Xue F. (2019). A novel approach for imaging of thoracic outlet syndrome using contrast-enhanced magnetic resonance angiography (CE-MRA), short inversion time inversion recovery sampling perfection with application-optimized contrasts using different flip angle evolution. Med. Sci. Monit..

[6] Weaver M., Lum Y. (2017). New Diagnostic and Treatment Modalities for Neurogenic Thoracic Outlet Syndrome. Diagnostics.

[7] Povlsen S., Povlsen B. (2018). Diagnosing thoracic outlet syndrome: Current approaches and future directions. Diagnostics.

[8] Masocatto N.O., Da-Matta T., Prozzo T.G., Couto W.J., Porfirio G. (2019). Thoracic outlet syndrome: A narrative review. Revista do Colégio Brasileiro de Cirurgiões.

[9] Hixson K., Horris H., Valovich-McLeod T., Welch-Bacon C. (2017). The Diagnostic Accuracy of Clinical Diagnostic Tests for Thoracic Outlet Syndrome. J. Sport Rehabil..

[10] Dessureault-Dober I., Bronchti G., Bussières A. (2018). Diagnostic Accuracy of Clinical Tests for Neurogenic and Vascular Thoracic Outlet Syndrome: A Systematic Review. J. Manip. Physiol. Ther..

[11] Jones M.R., Prabhakar A., Viswanath O., Urits I., Green J.B., Kendrick J.B., Brunk A.J., Eng M.R., Orhurhu V., Cornett E.M. (2019). Thoracic Outlet Syndrome: A Comprehensive Review of Pathophysiology, Diagnosis, and Treatment. Pain Ther..

[12] Gillard J., Pérez-Cousin M., Hachulla É., Remy J., Hurtevent J.F., Vinckier L., Thevénon A., Duquesnoy B. (2001). Diagnosing thoracic outlet syndrome: Contribution of provocative tests, ultrasonography, electrophysiology, and helical computed tomography in 48 patients. Jt. Bone Spine.

[13] Likes K., Rochlin D.H., Salditch Q., Dapash T., Baker Y., Deguzman R., Selvarajah S., Freischlag J.A. (2014). Diagnostic accuracy of physician and self-referred patients for thoracic outlet syndrome is excellent. Ann. Vasc. Surg..

[14] Hardy A., Pougès C., Wavreille G., Behal H., Demondion X., Lefebvre G. (2019). Thoracic Outlet Syndrome: Diagnostic Accuracy of MRI. Orthop. Traumatol. Surg. Res..

[15] Molina J.E., D’Cunha J. (2008). The vascular component in neurogenic-arterial thoracic outlet syndrome. Int. J. Angiol..

[16] Özcan H.N., Kara M., Özcan F., Bostanoǧlu S., Karademir M.A., Erkin G., Özçakar L. (2011). Dynamic doppler evaluation of the radial and ulnar arteries in patients with carpal tunnel syndrome. Am. J. Roentgenol..

[17] Hartley C., Reddy A., Madala S., Entman M., Taffet G. (2010). Feasibility of dual Doppler velocity measurements to estimate volume pulsations of an arterial segment. Ultrasound Med. Biol..

[18] Demondion X., Vidal C., Herbinet P., Gautier C., Duquesnoy B., Cotton A. (2006). Ultrasonographic assessment of arterial cross-sectional area in the thoracic outlet on postural maneuvers measured with power Doppler ultrasonography in both asymptomatic and symptomatic populations. J. Ultrasound Med..

[19] Makaryus A.N., Makaryus J.N., Figgatt A., Mulholland D., Kushner H., Semmlow J.L., Mieres J., Taylor A.J. (2013). Utility of an advanced digital electronic stethoscope in the diagnosis of coronary artery disease compared with coronary computed tomographic angiography. Am. J. Cardiol..

[20] Sztajzel J.M., Picard-Kossovsky M., Lerch R., Vuille C., Sarasin F.P. (2010). Accuracy of cardiac auscultation in the era of Doppler-echocardiography: A comparison between cardiologists and internists. Int. J. Cardiol..

[21] Takahashi O., Shimbo T., Rahman M., Musa R., Kurokawa W., Yoshinaka T., Fukui T. (2006). Validation of the auscultatory method for diagnosing peripheral arterial disease. Fam. Pract..

[22] Carmo G., Mandil A., Nascimento B.R., Arantes B.D., Bittencourt J.C., Falqueto E.B., Ribeiro A.L. (2009). Can we measure the ankle—Brachial index using only a stethoscope? A pilot study. Fam. Pract..

[23] Jou L.D., Mawad M.E. (2010). Indirect measurement of aneurysm wall thickness using digital stethoscope. Neurol. Res..

[24] Van Der Hoeven N.V., Van Den Born B.J.H., Van Montfrans G.A. (2011). Reliability of palpation of the radial artery compared with auscultation of the brachial artery in measuring SBP. J. Hypertens..

[25] Whiting P., Rutjes W., Westwood M., Mallet S., Deeks J., Reitsma J., Leeflang M.M.G., Sterne J.A.C., Bossuyt P.M.M. (2011). QUADAS-2: A Revised Tool for the Quality Assessment of Diagnostic Accuracy Studies. Ann. Intern. Med..

[26] Mokkink L.B., Terwee C.B., Patrick D.L., Alonso J., Stratford P.W., Knol D.L., Bouter L.M., de Vet H.C.W. (2010). The COSMIN checklist for assessing the methodological quality of studies on measurement properties of health status measurement instruments: An international Delphi study. Qual. Life Res..

[27] Perloff D., Grim C., Flack J., Frohlich E.D., Hill M., McDonald M., Morgenstern B.Z. (1993). Human Blood Pressure Determination by Sphygmomanometry. Circulation.

[28] Endres H.G., Hucke C., Holland-Letz T., Trampisch J. (2006). A new efficient trial design for assessing reliability of ankle-brachial index measures by three different observer groups. BMC Cardiovasc. Disord..

[29] Chesbro S.B., Asongwed E.T., Brown J., John E.B. (2011). Reliability of doppler and stethoscope methods of determining systolic blood pressures: Considerations for calculating an ankle-brachial index. J. Natl. Med. Assoc..

[30] Kaufmann C., Jacomella V., Kovacicova L., Husmann M., Clemens R.K., Thalhammer C., Amannvesti B. (2013). Predictive value of auscultation of femoropopliteal arteries. Swiss Med. Wkly..

